# 

**Published:** 1887-07-16

**Authors:** 


**The Hospital.**

**An Institution, Family, and Parochial Journal of Hospitals, Asylums, and all Agencies for the Care of the Sick, Criticism and News.**

SATURDAY, JULY 16TH, 1887.

### Notice to Advertisers.

*To insure prompt insertion, all Advertisements for* The Hospital *must be sent in direct to A. P. Watt,* 2, *Paternoster Square, E.C.*

### The Literary Prize.

A prize of **Two Guineas** will be given every month for the best story or incident based upon hospital, or asylum, or student life, or any incident connected with the professions of medicine or nursing.

*** The Children's Corner and Amusements with Profit will be found on page vi of this week's issue.

### THE IN- AND OUT-PATIENTS' HOSPITAL DIARY.

This Diary is so arranged as to make some portion of it appear every week, and the whole in each monthly part. It has been very difficult to compile, and corrections will at all times be gratefully received. It has been suggested that similar information about the larger Provincial and Scotch Hospitals would be useful to many readers. We should be glad to hear if this is felt to be the case.

### GENERAL HOSPITALS.—*continued.*

| Hospitals and Terms of Admission. | Physicians, Surgeons, and Medical Officers, with Days and Hours of Attendance. |
|---|---|
| St. Mary's Hospital, Cambridge Place, Paddington, W. Sec., Mr. Pietro Michelli. In-patients are admitted by letter of recommendation. Urgent cases and accidents free at all times. Out-patients require a letter. Electrical treatment Tu. and F. 2 | *In-Physicians*, Sir Edward Sieveking, Tu. F. 1.45; Drs. Broadbent, M. Th. 1.45; Cheadle, W. 1.45, S. 9.30. *In-Surgeons*, Messrs. Norton, M. Th. 1.45; Owen, Tu. F. 1.45; H. Page, W. S. 1.45. *Out-Physicians*, Drs. D. B. Lees, W. S.; S. Phillips, M. Th; R. Macguire, Tu. F. Hour, 1.30. *Out-Surgeons*, Messrs. W. Pye, M. Th.; A. J. Pepper, Tu. F.; A. Q. Silcock, W. S. Hour, 1.30 |
| St. Thomas's Hospital, Westminster Bridge Road, S.E. Steward, Mr. F. Walker. Accidents and emergencies free at all times. Other cases by Governor's letter, but, where unobtainable, application should be made to the Steward by letter, or personally between 10 and 4. Vaccination on W. at 11.30 | *In-Physicians*, Drs. Bristowe, Tu. F.; Stone, M. Th.; Ord, M. Th.; J. Harley, Tu. F.; Gervis, Tu. F. Hour, 2. *In-Surgeons*, Messrs. Sydney Jones, Tu. F.; Croft, M. Th.; Sir W. MacCormac, M. Th. Hour, 2. *Out-Physicians*, Drs. Payne, Tu. F.; Sharkey, M. Th.; Gulliver, W. S. Hour, 1.30. *Out-Surgeons*, Messrs. Clutton, Tu. F.; Anderson, W. S.; Pitts, M. Tu. Hour, 1.30 |
| Tottenham Training Hospital, Tottenham, N. Director, Dr. Laseron. Free. Accidents and emergencies admitted at all times | *In-Physician*, Dr. Rasch, Tu. F. 3 to 5. *In-Surgeons*, Drs. Lichtenberg, M. Th. 3 to 5; E. H. May, M. Th. 3 to 5. *Out-Surgeon*, Dr. Lloyd Smith, M. W. 11 to 2; *Men* only, W. 7 to 8 p.m. |
| University College Hospital (or North London), Gower Street, W.C. Sec., Mr. N. H. Nixon. Accidents and emergencies free. All other cases by Governor's letter of recommendation | *In-Physicians*, Drs. W. Fox, Tu. Th. 2, S. 9.30; Ringer, M. W. F. 1.30; Bastian, M. 2.30, W. 10, F. 2.30; F. Roberts, Tu. 1.15, Th. 1.30, S. 9.30. *In-Surgeons*, Messrs. B. Hill, Tu. 2.15, W. 2, F. 2.15; C. Heath, M. W. F. 2; M. Beck, Tu. Th. S. 2. *Out-Physicians*, Drs. Gowers, W. S.; Poore, Tu. F.; Barlow, M. Th. Hour, 1.30. *Out-Surgeons*, Messrs. Barker, M. Th.; Godlee, Tu. F.; Horsley, W. S. Hour, 1.30 |
| West London Hospital, Hammersmith Road, W. Sec. and Supt., Mr. R. J. Gilbert. Accidents and emergencies free at all times. Other cases require a letter of recommendation. New cases must attend at 1.30 | *In-Physicians*, Drs. G. Rogers, D. W. Hood, F. Drewitt. *In-Surgeons*, Messrs. C. Keetley, F. Edwards, W. B. Clarke. *Out-Physicians*, Drs. Herringham, M. Th.; Ball, Tu. F.; S. Taylor, W. S. Hour, 2. *Out-Surgeons*, Messrs. Ballance, Tu. F.; Weiss, M. Th.; Wainewright, W. S. Hour, 2 |
| Westminster Hospital, Broad Sanctuary, S.W. Sec., Mr. S. M. Quennell. Accidents and urgent cases free at all times. Other cases require a letter of recommendation | *In-Physicians*, Drs. Sturges, M. Th. S.; Allchin, M. Tu. F.; Donkin, Tu. W. S. Hour, 1.30. *In-Surgeons*, Messrs. Cowell, M. Tu. Th.; Davy, M. Tu. F.; Macnamara, M. W. S. Hour, 1.30. *Out-Physicians*, Drs. De H. Hall, M. Th.; A. H. Bennett, Tu. F.; Murrell, W. S. Hour, 1.30. *Out-Surgeons*, Messrs. Cooke, M. Th.; Bond, Tu. F.; Barrow, W. S. Hour, 1.30 |

### II.—SPECIAL HOSPITALS.

### CANCER.

| | |
|---|---|
| Cancer Hospital, Brompton, S.W. Sec., Mr. W. H. Hughes. Entirely free to both in- and out-patients | *In- and Out-Surgeons*, Drs. A. Marsden, W.; H. Snow, M.; F. A. Purcell, F.; Messrs. F. B. Jessett, W.; C. Stonham, Th.; C. Jennings, Tu.; W. H. Elam, S. Hour, 2 |
| London Hospital, Whitechapel Road, E. | Daily, 1.30. *See* General Hospitals |
| Middlesex Hospital, Berners Street, W. Admission free | *In-Surgeons*, Messrs. Hulke, Lawson, and Morris. *Out-Surgeon*, Mr. Morris, Th. 1.30 |
| St. Saviour's Hospital, Osnaburg Street, N.W. Sec., Mr. E. Poitiers. By Governors' letters | *In- and Out-Physician*, Dr. A. Kennedy, 10.30 and 4 daily |

### CHILDREN'S DISEASES.

| Hospitals and Terms of Admission. | Physicians, Surgeons, and Medical Officers, with Days and Hours of Attendance. |
|---|---|
| Alexandra Hospital for Children, 18, Queen Sq., W.C. Sec., Mrs. Marsh. For hip disease | *Physician*, Dr. Steavenson. *Surgeons*, Messrs. H. Marsh Tu. F.; A. Bowlby, M. Th. Hour, 4.30 (No out-patients) |
| All Saints' Children's Hospital, 4, Margaret Street, W. For chronic joint diseases | *Surgeon*, Mr. N. Smith (No out-patients) |
| BelgraveHosp. for Children, 77 & 79, Gloucester Street, Pimlico. Hon. Secs., Capt. Stopford, and Rev. J. Storrs. By letters; accidents free | *In- and Out-Physicians*, Drs. W. Hope and W. Ewart, M. Th. 9. *In- and Out-Surgeons*, Messrs. C. T. Dent and Margerison, Tu. F. 9 |
| Charing Cross Hospital, West Strand, W.C. | *Out-Physician*, Dr. Nursby, W. S. 1.30 |
| Cheyne Hospital for Sick and Incurable Children, 46, Cheyne Walk, Chelsea, S.W. Sec., Miss D. Evans | *Physician*, Dr. G. V. Poore. *Surgeons*, Messrs. T. P. Bartlett and J. Macready attend alternate days in afternoons. (No out-patients) |
| East London Hospital for Children and Dispensary for Women, Glamis Road, Shadwell, E. Sec., Mr. Ashton Warner. By Governor's letter. Urgent cases and accidents are admitted free | *In-Physicians*, Drs. Donkin, M. Th.; Eustace Smith, T. F.; Crocker, F. *In-Surgeons*, Messrs. A. Cæsar, R. W. Parker, W. F. *Out-Physicians*, Drs. Crocker, M.; Dawson Williams, Tu. Th.; Coutts, W. F. Hours, 1 and 3. *Out-Surgeon*, Mr. Dunn, Tu. F. 1, 3 |
| Evelina Hospital for Sick Children, Southwark Bridge Road, S.E. Sec., Mr. T. S. Chapman. Free, but Governor's letter takes precedence. In-patients (boys) between 2 and 10, (girls) between 2 and 12 | *In-Physicians*, Drs. F. Taylor and J. Goodhart. *In-Surgeons*, Messrs. H. G. Howse and R. C. Lucas. *Out-Physicians*, Drs. N. Tirard, M. Th. 9; F. Willcocks, Tu. F. 9. *Out-Surgeons*, Messrs. C. J. Symonds, S. 9; G. H. Makins, W. 9 |
| German Hospital, Dalston Lane, Dalston, E. | *In-Physician*, Dr. Rasch, M. Th. 9 |
| Great Northern Central Hospital, Caledonian Rd. N. | *Physicians*, Drs. Murray and Barnes W. 2.30 |
| Grosvenor Hospital for Women and Children, Vincent Sq., S.W. Hon. Secs., Mr. A. J. Ram and Miss Day. By letter or payment | *In- and Out-Physicians*, Drs. R. Gibbons and Steavenson, Tu. W. Th. S. 2. *In- and Out-Surgeons*, Messrs. Smythe and Parker, Tu. W. Th. S. 2 |
| Hospital for Sick Children, 48 and 49, Great Ormond St., W.C. Sec., Mr. S. Whitford. By Governors' letters. If without letter, the case of the applicant is investigated. In-patients between 2 and 12; Out-patients under 12 | *In-Physicians*, Drs. W. Cheadle, Tu. F.; Sturges, W. S.; Barlow, M. Th. Hours, 9 to 11. *In-Surgeons*, Messrs. H. Marsh, W. S.; E. Owen, W. S. Hours, 9 to 11. *Out-Physicians*, Drs. R. J. Lee and Abercrombie, M. Th.; Lubbock and Money, Tu. F. Hours, 8.30 to 10. *Out-Surgeons*, Messrs. Morgan and Pitts, W. S. 8.30 to 10 |
| King's College Hospital, Portugal Street, W.C. | *In- and Out-Physician*, Dr. T. C. Hayes, W. F. 1 |
| Middlesex Hospital, Berners Street, W. Free. Children under 3 | *Out-Physician*, Dr. Duncan, W. S. 1.30 |
| North Eastern Hospital for Children, Hackney Road, E. Sec., Mr. A. Nixon. By free ticket or small payment | *In- and Out-Physicians*, Drs. W. Cayley, F. Turner, C. Semple, and W. Pasteur, M. W. Th. S. 1.30. *In- and Out-Surgeons*, Messrs. Waren Tay and Pollard, M. 1.30, F. 9 |
| Paddington Green Children's Hosp., Paddington Green. Sec., Mr. W. H. Pearce. Admission free; boys under 12; girls under 14 | *In- and Out-Physicians*, Drs. T. Lauder Brunton, Ogilvie, Tu. F.; G. Laycock, M. Th.; S. Phillips, W. S. Hour, 9.15. *In- and Out-Surgeons*, Messrs. W. W. Cheyne, M., S. Boyd, F. Hour, 9.15 |
| Royal Hospital for Children and Women, Waterloo Bridge Road, S.E. Sec., Mr. R. G. Kestin. Out-Patients pay 1*d.* on each attendance. Boys not admissible, as out-patients, over 12, or, as in-patients, over 8 | *In-Physicians*, Drs. Park, M. Th.; Gulliver, Tu. F.; Price, W. S. Hour, 2. *In-Surgeon*, Mr. W. H. A. Jacobson, M. Th. Hour, 2. *Out-Physicians*, Drs. Park, M. Th.; Dakin, W. F.; Hadden, Tu. S. Hour, 2. *Out-Surgeon*, Mr. E. O. Day, W. 2 |
| St. Mary's Hospital, Cambridge Place, Paddington | *Out-Physician*, Dr. M. Handfield Jones, M. Th. 1.30 |
| St. Thomas's Hospital, Westminster Bridge Road, S.E | *Out-Physician*, Dr. Cory, W. S. 1.30 |

### The Children's Corner.

**Fourth Quarterly Prize Competition.**

Began 2nd July, 1887 ; ends 24th Sept., 1887.

PRIZES.

FOR MOST CORRECT ANSWERS TO PUZZLES.

| | | | |
|---|---|---|---|
| 1st .. | .. Thirty Shillings | 3rd .. | .. Ten Shillings |
| 2nd .. | .. Twenty " | 4th .. | .. Five " |

FOR NEATEST ANSWERS TO PUZZLES.

Five Shillings.

FOR BEST LITTLE LETTERS.

Ten Shillings.

**Puzzles—Third Week.**

No. 9. BURIED TOWNS AND COUNTIES. 1. Are you going to spend your holidays at Brighton or Folkestone? 2. I wonder by which train you will start? 3. I always drink water for dinner. 4. Here is a bed for a dolly. 5. Do witches hire brooms to ride to the sky?

No. 10 The following letters, arranged in their proper order, will give a well-known proverb:—R a a s s i i l l l l n o o h d t t t t t e g g.

No. 11. TREE PUZZLE. 1. Which is the tree which is nearest the sea? 2. The Chronological tree? 3. The tree that is most warmly clad?

No. 12. CHARADE.

If you and I together fought,
Whoever won, my *first* would be.
And now attend! two vowels add,
A famous *Queen* you then will see.

**Letters—Third Week.**

The Letter-subject for next week will be *Boating.*

**Results of First Week—Puzzles.**

No. 1. ENIGMA. Straw-berry.

No. 2. ARITHMETICAL PUZZLE. There are three solutions of this puzzle:—9¼, 9½, or 9¾.

No. 3. CONUNDRUM. Why is Eau de Cologne like a foreign letter? Because it is sent (scent) from abroad.

No. 4. GEOGRAPHICAL CHARADE The town is Bridgewater.

*All right*—Poodle, The Eda, A. C. K., A. G. S. McCulloch, Umslopogaas, Scrooge, Ellie; *Three right*—Princess Ida, Toby, Skye, Agatha, Bohemian Girl, Mikado, Alsie, Ossian, Mount Edgcumbe; *Two right*—Socrates, Violin, Edith.

**Letters.**

I am very glad to welcome several new little friends this week, and they, in common with my usual correspondents, have all something pleasant to say on the subject of how a half-holiday can be spent. Here is a very good letter from Toby, aged 14, which will interest all children who are fond of dogs:—

THE HOME FOR LOST DOGS.

My favourite way to spend a half-holiday is to take the train from Chelsea Station, which is quite close to our house, to Battersea. From this station a short walk brings you to Battersea Park, with its shoals of seats, and beautiful ornamental water, to say nothing of the flower-beds, which are exquisitely laid out with choice flowers. But passing through, you come to what I find the centre of amusement, "The Home for Lost Dogs." On entry a notice fastened to the wall requests you to communicate with the secretary, who is in a little side office. You tell him that you wish to look over the Home, and he immediately gives permission, whereupon you pass through a door into an open yard, and are saluted with a volley of whines and barks. On turning a corner, you see a great number of dog-kennels, or rather runs, some of which are partly covered over, and some are placed in rows in good-sized sheds. There are also open enclosures, strongly railed in, in which the dogs take exercise. One of these enclosures is provided with a tank of water let into the asphalted flooring, and in this the dogs that are fond of the water revel. On the last occasion that I visited this Home, I noticed especially a large brown retriever, with most of its coat clipped, which was very savage, and whenever anyone approached it, a savage bark was the result. The man in charge told us that he had over two hundred dogs in the buildings at that time. He pointed out to us two very pretty little puppies which were for sale. They had been at the Home for some days when we saw them. There is also a system of punishment for the refractory dogs, namely, solitary confinement. We saw a large black retriever in a pen quite by himself, with a board on the gate, which had "Three Days" written on it. The man to'd us hat it had shown signs of a bad temper, and that it was given to biting, hence the punishment. A great many of the dogs are really very fine ones, and I am sure that if anybody is at all fond of dogs, and has not visited this Home, he will find it well worth a visit on any half-holiday he has to spare.

I am delighted to welcome Etta again, and to hear that her hand is well enough to allow her to continue her weekly letter. Also that Skye has persuaded her brother Ossian to join us, and he sends me a very nice letter. Socrates and Mount Edgcumbe both send well-written and thoughtful letters, which I cannot insert, as they are written on both sides of the paper, a very common mistake with new competitors. Here are two letters from Poodle and Rambler, each showing an enjoyable way of spending a half-holiday in the country.

HAYMAKING.

Poodle, aged 14, writes from Worthing:—

I think that my favourite way of spending a half-holiday is to go haymaking. A short time ago I had great fun in a hay-field. There were some other children there, so my brother and I made a big nest, and we got the others to go inside, and then ran against it, and pushed it down all over them, and almost buried them. Then we had tea, and after that it was time to leave. We had to drive four and a half miles home in the evening, which was very nice, it being so cool. I enjoyed myself very much, and I think my brother and sisters did too.

BLACKBERRIES AND BLACKBERRYING.

Rambler, aged 13, writes from Wood's Farm, Gloucester:—

My most enjoyable half-holidays are always spent in the late weeks of September and in the early part of October, for then it is not so hot and tiring to walk about. I really think that nothing could be more delightful than climbing Robin Hood Hill in company of other young people on a cool day at this time, for then the blackberries are on the bushes, and we can pick and eat at our leisure but of course we don't forget those at home, but usually bring back large baskets full. We have little difficulties in all enjoyments, and those under "blackberry gathering" are numerous scratches and thorns in our hands, which we find out more on the next day. I delight very much on these occasions to take provisions with us, and there is a dear old woman at the foot of the hill who is always pleased to prepare tea for us, and then we sit in a very comfortable manner on the grass and satisfy our terrible appetites.

One mark each:—Toby, Rambler, Poodle, Mount Edgcumbe, Ossian, Socrates, Etta, Bohemian Girl, Eric of the Eda, Princess Ida, Ellie, A. G. S. McCulloch, Skye, Edith.

### Amusements with Profit.

### PRIZE COMPETITIONS.

**Quarterly Prize Competition.**

Began 23rd April; ends 16th July.

**A Prize of Three Guineas**

will be awarded to the Competitor who shall have sent in the largest number of correct or best answers. No one will be permitted to win the Three Guinea Prize twice in any one year, but we shall award annually an additional

**Prize of Five Guineas**

to the competitor who shall have sent in the largest number of correct or best answers during the twelve months.

**No. 24. Double Acrostic.**

My second lately of my first
Has been the universal theme;
Though cavillers have done their worst,
It has surpassed the fondest dream.

1. Two and yet one, divided *I* expire,
2. Who'd of thy fair valley ever tire?
3. Sacred to dull November's early days,
4. Victoria's as Elizabeth's we praise.
5. *This* to your clemency for faults I make,
6. A cipher *I* if you my value take.
7. This patriots still are called for old sake's sake.

**No. 25. Algebraic Problem.**

There is a certain number which, being divided by the sum of its digits, gives for the quotient the first digit; but, on the digits being inversed, and the number thus formed divided by the sum of the digits, the quotient is 7. Required the number.

**Answers.**

No. 21. DOUBLE ACROSTIC.

B al L

E ffend I

A go G

C oac H

O liphan T

N are S

Correct answers have been received from Boss, Gretta, Aralc, K. M., Common Sense, Mater, Canice, Letheàdor, Dawnlover, A. M. E., Mrs. Bedingfeld.

No 22. LOYALTY.

We have received many definitions of Loyalty; all showing a thoughtful and intelligent appreciation of the sentiment, whether taken in its fullest and widest sense, or treated from a more limited point of view.

Answers have been received from K. M., Dawnlover, Mater, Letheàdor, Aralc, Common Sense, Canice, Ellice, A. M. E. Gretta.

